# Bibliometric and visual analysis of intestinal flora and immunity

**DOI:** 10.1097/MD.0000000000036575

**Published:** 2024-01-26

**Authors:** Kaidi Nie, Tingting Deng, Jie Wang, Luming Qi, Nannan Liu, Zhixuan Chen, Lina Xia

**Affiliations:** a State Administration of Traditional Chinese Medicine, Key Laboratory of Traditional Chinese Medicine Regimen and Health, Chengdu University of Traditional Chinese Medicine, Chengdu, Sichuan, China; b School of Health Preservation and Rehabilitation, Chengdu University of Traditional Chinese Medicine, Chengdu, Sichuan, China; c Key Laboratory of Traditional Chinese Medicine Regimen and Health of Sichuan Province, Chengdu, Sichuan, China.

**Keywords:** bibliometrics, gastrointestinal microbiome, immunity

## Abstract

**Background:**

The gut microbiota and its stability have important relationships with immunity. However, bibliometric analysis in this field is underdeveloped. This study aims to visualize publications related to the gut microbiota and immunity to identify research frontiers and hotspots, providing references and guidance for further research.

**Methods:**

Gut microbiota and immunity data were retrieved from the Web of Science Core Collection database, and Microsoft Excel, Scimago Graphica and VOSviewer software were used to analyze publication output trends, the most productive countries/regions, journals, authors, co-cited references, and keywords.

**Results:**

This study analyzed 16,611 publications, including 10,865 articles and 5746 reviews, and found a continuous increase in publications related to gut microbiota and immunity since 2013. We identified 62,872 authors contributing to this field from 2144 journals and 9965 organizations/institutions in 145 countries/regions. The top publisher with the highest output is University of California System with 525 papers. Among these journals, the top 3 most prolific journals are Frontiers in Immunology, Frontiers in Microbiology, and PLOS ONE. The literature with the highest citation frequency is published in Science and has been cited 3006 times by Patrick M. Smith and others.

Gut microbiota research hotspots include gut microbiota inflammation, immune response, inflammatory bowel diseases (IBDs), and microbiota tumors. The gut microbiota and its microbial homeostasis play critical roles in immune reactions, inflammation, and even tumors and IBDs.

Current research on gut microbiota and immunity is a popular field. Previous studies have shown that the gut microbiota and its microbial species have important effects on maintaining human health, immune function, inflammation, tumorigenesis, and IBDs. Understanding the roles of microbial communities and specific bacterial species as well as their interactions with humans has led to numerous discoveries that provide unique opportunities for exploring human health and future research.

**Conclusion:**

This study used bibliometric and visualization analysis to identify the development trends and hotspots of publications related to the gut microbiota and immunity. The findings of this study provide valuable insights into the emerging trends and future directions in this field.

## 1. Introduction

The gut microbiota is crucial for human health, and its changes are closely related to the occurrence and development of many systemic diseases. With continuous changes in lifestyle and eating habits, the gut microbiota is undergoing changes, and the disease spectrum of humans is also undergoing significant changes. More and more studies are dedicated to clarifying the impact of the gut microbiota on human health and disease. The gut microbiota, as a “microbial organ,” directly regulates the body health status through cell components such as lipopolysaccharides and metabolites such as trimethylamine oxide and short-chain fatty acids, and also affects the body immune system through bacteria and their products.^[1–6]^ At the same time, the gut microbiota and its microbial homeostasis are closely related to inflammation, the occurrence of tumors, and the prevention of cardiovascular diseases.^[7–16]^How the gut microbiota regulates the occurrence and development of certain systemic diseases by modulating the immune system is currently a research hotspot. However, these studies have not been summarized and analyzed as a whole. Bibliometric analysis has been widely used to investigate the development trends and major findings of scientific research by quantitatively analyzing previous scientific literature. In our study, bibliometric analysis was conducted on the gut microbiota and immunity to showcase the current research status, draw visual maps, explore trends and hotspots in the field, and provide references for future research.

## 2. Materials and methods

### 2.1. Search strategy

The Web of Science Core Collection (WoSCC) is a reputable citation-based database with powerful indexing capabilities widely used for bibliometric analysis.^[17,18]^ In this study, we opted to download literature related to the gastrointestinal microbiome and immunity from WoSCC. We retrieved articles related to the gastrointestinal microbiome using keywords such as “Gastrointestinal Microbiome” or “Gastrointestinal Microbiomes,” and we obtained immune-related articles using keywords like “Immunity” or “Immune Process” (see Table 1 for details). We combined these 2 sets of keywords for a comprehensive search. The search results were filtered to include only research papers and reviews, written in English, published between 2013 and 2022.

**Table 1:** The search term TS are shown in the following table.

	TS	
Intestinal flora; Gastrointestinal Microbiome. Gastrointestinal Microbiomes. Microbiome, Gastrointestinal; Gut Microbiome. Gut Microbiomes. Microbiome, Gut; Gut Microflora. Microflora, Gut; Gastrointestinal Flora; Flora, Gastrointestinal; Immunity. Immune Process;	Gut Flora; Flora, Gut. Gastrointestinal Microbiota: Gastrointestinal Microbiotas; Microbiota, Gastrointestinal; Gastrointestinal Microbial Community; Gastrointestinal Microbial Communities; Microbial Community, Gastrointestinal; Gastrointestinal Microflora; Microflora, Gastrointestinal; Gastric Microbiome Immune Processes; Process, Immune;	Gastric Microbiomes: Microbiome, Gastric; Intestinal Microbiome; Intestinal Microbiomes; Microbiome, Intestinal; Intestinal Microbiota; Intestinal Microbiotas; Microbiota, Intestinal; Intestinal Flora; Flora, Intestinal; Enteric Bacteria Immune Response; Immune Responses Response, Immune

### 2.2. Data collection and analysis

The data related to records of data collection and analysis were exported in the format of “full-text records and cited references.” Bibliometric analysis was conducted on the obtained publications, summarizing the characteristics of highly productive authors, institutions, countries/regions, journals, and the top 10 highly cited papers.

VOSviewer, a bibliometric tool for constructing and visualizing bibliometric maps, was utilized.^[19,20]^ Distances and colors represent the distribution of nodes in 2-dimensional space (association and time). The size of labels and circles indicates the significance of the content. In our study, VOSviewer 1.6.16 software was employed to analyze the complete co-authorship of authors, organizations, countries, journals, publications, and all keywords. The minimum number of occurrences set for the network was 5.

## 3. Results

### 3.1. Publication output and time trend

**Figure 1.**
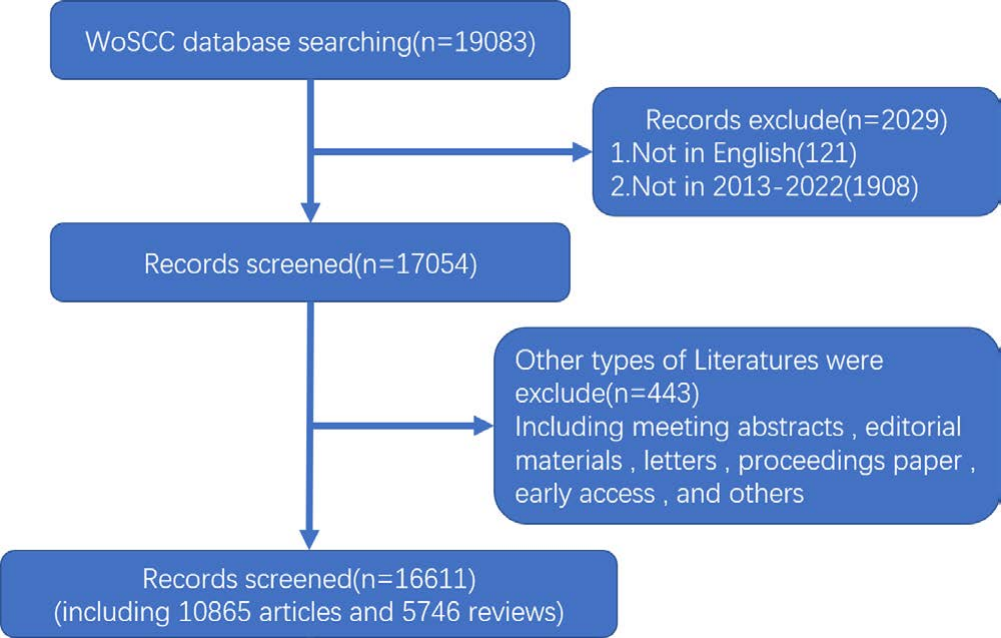
Flowchart of data filtration processing and excluding publications.

**Figure 2.**
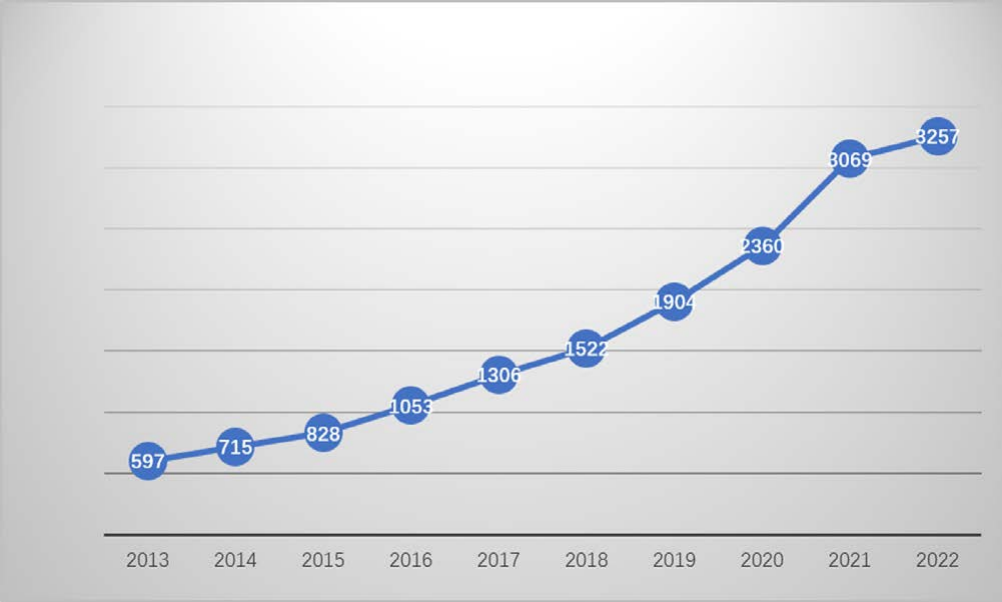
Annual publications quantitative distribution from 2013–2022.

A total of 19,083 papers were identified, and based on the selection criteria, 16,611 English articles published between 2013 and 2022 were included, comprising 10,865 research papers and 5746 reviews, as illustrated in Figure 1. Since 2013, there has been a significant upward trend in publications in this field. In the analysis of publications, the leading journal publishers are Frontiers in Immunology (787 publications) and Frontiers in Microbiology (500 publications), as shown in Figure 2. These data suggest that these 2 publishers are at the forefront in terms of research publication output in the fields of immunology and microbiology.

### 3.2. Author distribution

**Figure 3.**
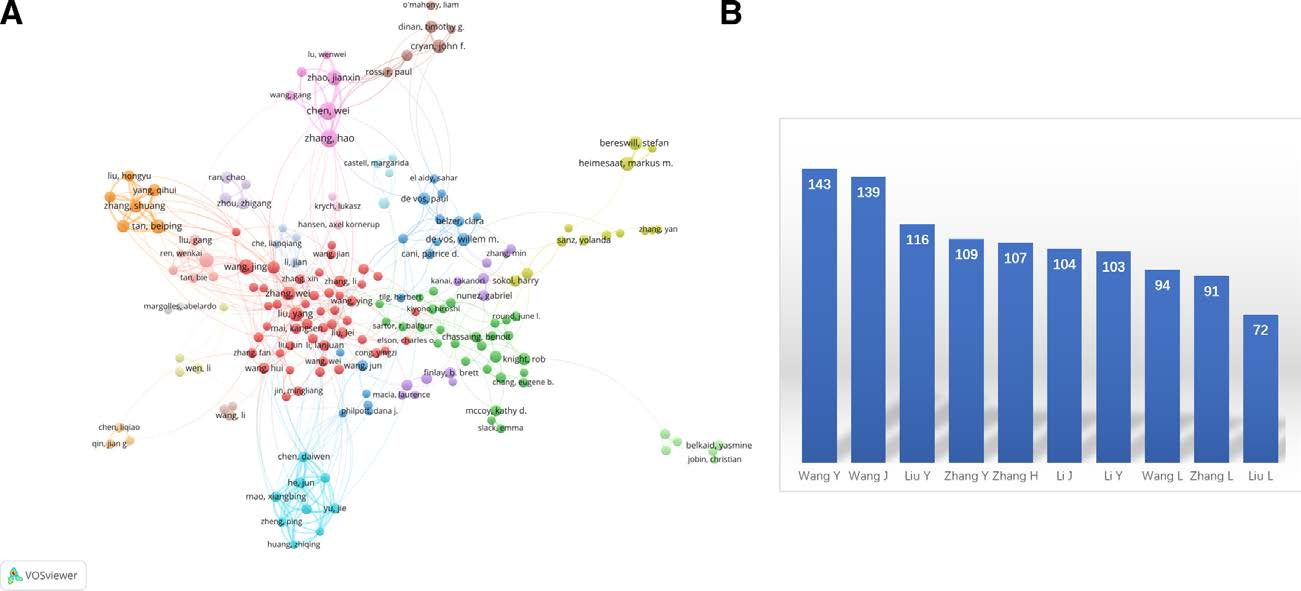
(A) The co-authorship map of authors. (B) Number of publications issued by authors.

A total of 62,872 authors have contributed to the field. The top 10 authors with the highest number of publications are as follows: Wang Ying from China, with 143 published papers; followed by Wang Jie with 139 papers, Liu Ying with 116 papers, Zhang Yi with 109 papers, Zhang Hua with 107 papers, Li Jie with 104 papers, Wang Lin with 94 papers, Zhang Lin with 91 papers, and Liu Lin with 72 papers. The collaborative relationships among authors are depicted in Figure 3.

### 3.3. Distribution by country/region/institutions

The publications included in this study originate from approximately 9965 organizations across 145 countries/regions. The leading institutions in terms of publication count are University of California System, with 525 papers, followed by the Udice French Research Universities with 463 papers, Harvard University with 452 papers, the Ministry of Agriculture Rural Affairs with 439 papers, the Chinese Academy of Sciences with 373 papers, Institute National de la Sante et de la Recherche Medicale INSERM with 373 papers, Harvard Medical School with 326 papers, Centre National de la Recherche Scientifique CNRS with 260 papers, INRAE with 255 papers and Zhejiang University with 242 papers.

**Figure 4.**
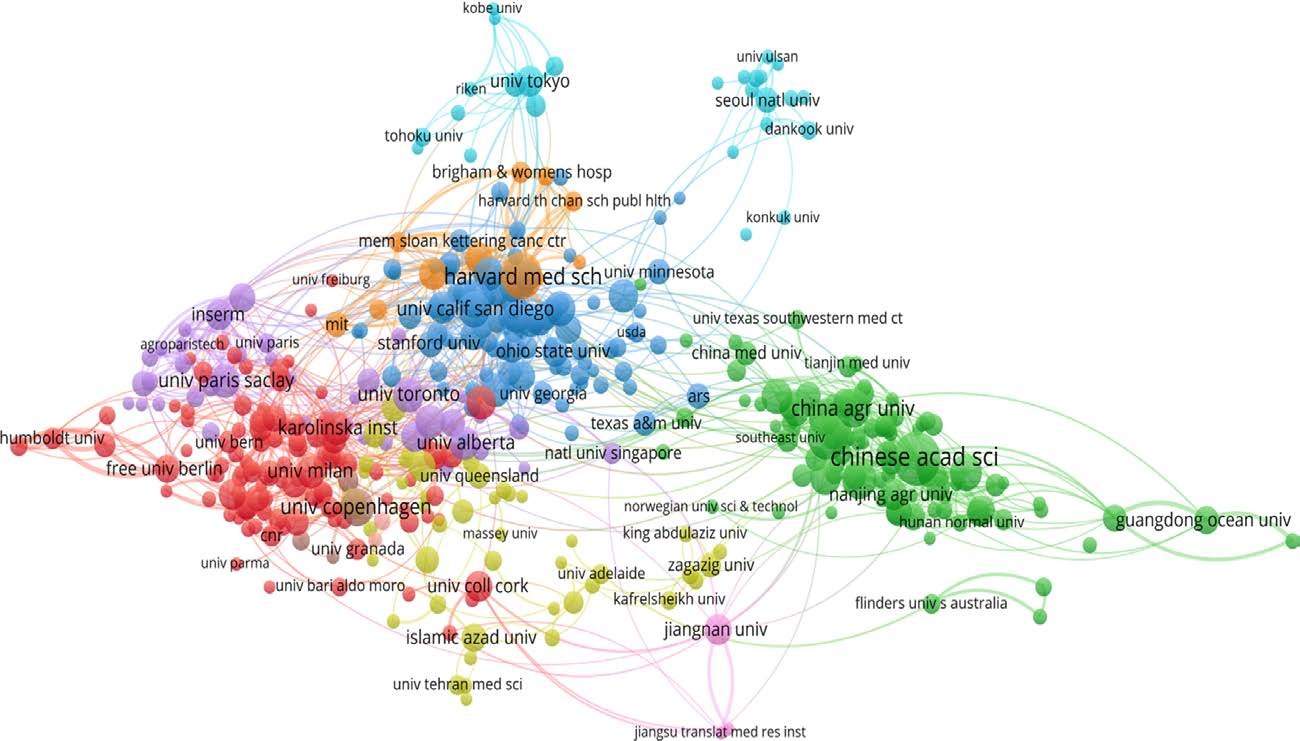
The co-authorship map of organizations. University of California System has published the most related papers (525 items).

**Figure 5.**
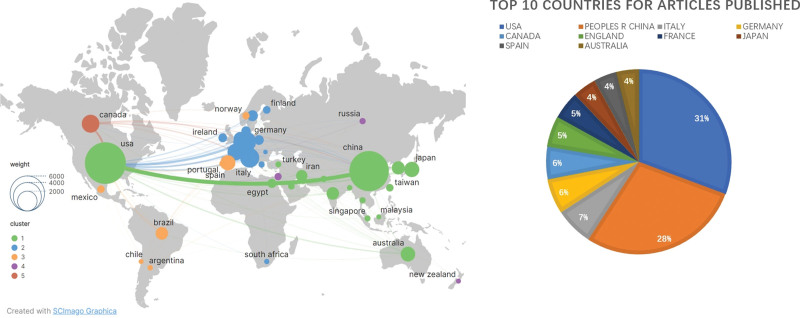
Ranking of published articles by countries.

The network visualization of institutions is depicted in Figure 4. In terms of countries/regions, the United States has the highest number of published papers with 4900 papers, followed by the People Republic of China with 4547 papers, Italy with 1063 papers, Germany with 982 papers, Canada with 908 papers, the United Kingdom with 867 papers, France with 780 papers, Spain with 651 papers, and Australia with 618 papers. The network visualization map of countries/regions is illustrated in Figure 5. The top 10 countries/regions and institutions with high productivity are summarized in Table 2.^[21–24]^

**Table 2:** Ranking of the top 10 countries, institutions, and authors.

Items	Publications					
	Ranking	Country	Number	Percentage	Citations	H-index
Country	1 2 3 4 5 6 7 8 9 10	USA PEOPLES R CHINA ITALY GERMANY CANADA ENGLAND FRANCE JAPAN SPAIN AUSTRALIA	4900 4547 1063 982 908 867 780 657 651 618	29.499 27.373 6.399 5.912 5.466 5.219 4.696 3.955 3.919 3.720	284290 95308 46518 46662 45819 47197 45789 30532 28083 30706	215 119 101 90 105 106 100 77 85 83
Institution	1 2 3 4 5 6 7 8 9 10	University of California system UDICE French research universities Harvard University Ministry of agriculture rural affairs Chinese academy of sciences Institut national de la sante et de la recherche medicale inserm Harvard medical school Centre national de la recherche scientifique Inrae Zhejiang university	525 463 452 439 373 373 326 260 255 242	3.161 2.787 2.721 2.643 2.245 2.245 1.963 1.565 1.535 1.457	46798 37745 52341 8811 10749 32655 37414 20052 21072 6060	101 92 99 45 57 85 78 64 63 40
Author	1 2 3 4 5 6 7 8 9 10	Wang Ying Wang Jie Liu Ying Zhang Yi Zhang Hua Li Jie Li Ying Wang Lin Zhang Lin Liu Lin	143 139 116 109 107 104 103 94 91 72	0.861 0.837 0.698 0.656 0.644 0.626 0.620 0.566 0.548 0.433	2434 3487 1743 4802 1905 2151 3042 1557 1823 1113	27 28 22 21 27 24 27 20 23 18

### 3.4. Publication distribution

**Figure 6.**
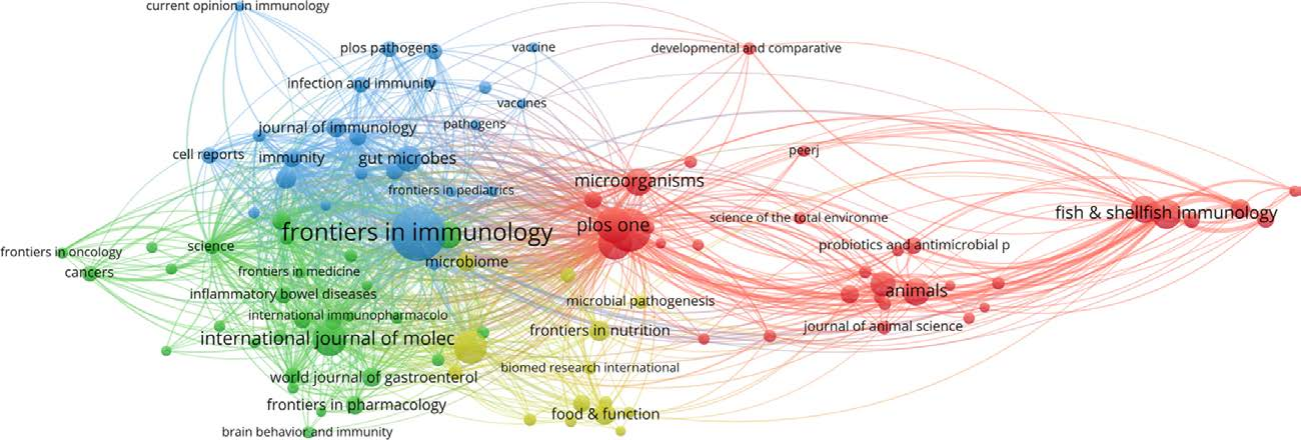
Visualization knowledge maps of citation. The size of the nodes represents the counts of citations. The distance between the 2 nodes indicates their correlation.

All publications are from 2144 journals. Among them, “FRONTIERS IN IMMUNOLOGY” ranks first with 787 papers, “FRONTIERS IN MICROBIOLOGY” ranks second with 500 papers, and “PLOS ONE” ranks third with 325 papers. The network visualization diagram of all journals is shown in Figure 6. The top 10 high-output publications by paper count are shown in Table 3.

**Table 3:** Ranking of the top 10 journals based on publications.

Ranking	Journal name	Counts	Percentage	Citations	IF (2023)	Quartile in category
1 2 3 4 5 6 7 8 9 10	FRONTIERS IN IMMUNOLOGY FRONTIERS IN MICROBIOLOGY PLOS ONE INTERNATIONAL JOURNAL OF MOLECULAR SCIENCES SCIENTIFIC REPORTS NUTRIENTS FISH SHELLFISH IMMUNOLOGY AQUACULTURE MICROORGANISMS ANIMALS	787 500 325 305 299 296 240 206 200 195	4.738 3.010 1.957 1.836 1.800 1.782 1.445 1.240 1.204 1.174	21692 14187 11340 6705 8814 8097 9096 4287 2666 1609	7.3 5.2 3.7 5.6 4.6 5.9 4.7 4.5 4.5 3.0	Q1 Q1 Q2 Q1 Q2 Q1 Q1 Q1 Q2 Q1

### 3.5. Analysis of highly cited literature

The characteristics of the top 10 highly cited literature^[25–34]^ are presented in Table 4. The 3 most frequently cited publications are as follows: the first article, titled “The microbial metabolites, short chain fatty acids, regulate colonic Treg cell homeostasis,” was published in the journal Science by Patrick M. Smith1, Michael R. Howitt1, Nicolai Paniko, et al^[31]^ This article discusses the regulation of colonic regulatory T cells (cTregs) expressing the transcription factor Forkhead box protein 3(Foxp3), which play a crucial role in limiting intestinal inflammation and rely on signals derived from the microbiota for their proper development and function.^[35–38]^

**Table 4:** Ranking of the top 10 highest cited references.

Ranking	Title	Total citations	Author	location	IF (2023)	Quartile in category
1 2 3 4 5 6 7 8 9 10	The Microbial Metabolites, Short-Chain Fatty Acids, Regulate Colonic T-reg Cell Homeostasis Commensal microbe-derived butyrate induces the differentiation of colonic regulatory T cells Gut microbiome influences efficacy of PD-1-based immunotherapy against epithelial tumors Metabolites produced by commensal bacteria promote peripheral regulatory T-cell generation Role of the Microbiota in Immunity and Inflammation Gut microbiome modulates response to anti-PD-1 immunotherapy in melanoma patients Commensal Bifidobacterium promotes antitumor immunity and facilitates anti-PD-L1 efficacy The Treatment-Naive Microbiome in New-Onset Crohn Disease Treg induction by a rationally selected mixture of Clostridia strains from the human microbiota Host microbiota constantly control maturation and function of microglia in the CNS	3006 2942 2644 2562 2469 2250 2089 1870 1796 1664	Patrick M. Smith Furusawa Yukihiro Routy Bertrand Nicholas Arpaia Belkaid Yasmine Gopalakrishnan V Sivan Ayelet Gevers Dirk Atarashi Koji Erny Daniel	USA Japan France USA USA USA USA USA Japan Germany	56.9 64.8 59.6 64.8 64.5 56.9 56.9 30.3 64.8 25.0	Q1 Q1 Q1 Q1 Q1 Q1 Q1 Q1 Q1 Q1

The researchers discovered that the beneficial impact of short chain fatty acids (SCFA) on SPF mice is attributed to their capacity to enhance the production of Regulatory T Cells producing Interleukin-10 (Foxp3 + IL-10) producing colonic regulatory T cells (cTregs) and increase cTreg proliferative capability, while also modifying cTreg G protein-coupled receptor 15(GPR15) expression.

A study published in the journal Science by Bertrand Routy, Emmanuelle Le Chatelier, Lisa Derosa^[25]^ demonstrated that the gut microbiome influences the effectiveness of Programmed cell death protein 1 (PD-1)-based immunotherapy against epithelial tumors. The article elucidates that immune checkpoint inhibitors (ICIs) targeting the PD-1/PD-L1 axis elicit sustained clinical responses in a significant subset of cancer patients. Furthermore, it was found that abnormal composition of the gut microbiome contributes to primary resistance to ICIs. To validate this, fecal microbiota transplantation (FMT) from ICI-responsive cancer patients into germ-free or antibiotic-treated mice ameliorated the antitumor effects of PD-1 blockade.

The third most cited publication, a review article titled “Role of the Microbiota in Immunity and Inflammation,” was authored by Yasmine Belkaid, Timothy W. Hand, and others,^[29]^ and published in the journal Cell. The article highlights the harmonious alliance between the microbial community and the host immune system, which induces protective responses against pathogens and maintains regulatory tolerance towards innocuous antigens. However, the dramatic increase in autoimmune and inflammatory diseases is attributed to the lack of diverse microbial communities required to establish balanced immune responses, primarily due to factors such as overuse of antibiotics and alterations in dietary patterns.

### 3.6. Cluster analysis of co-occurring keywords

**Figure 7.**
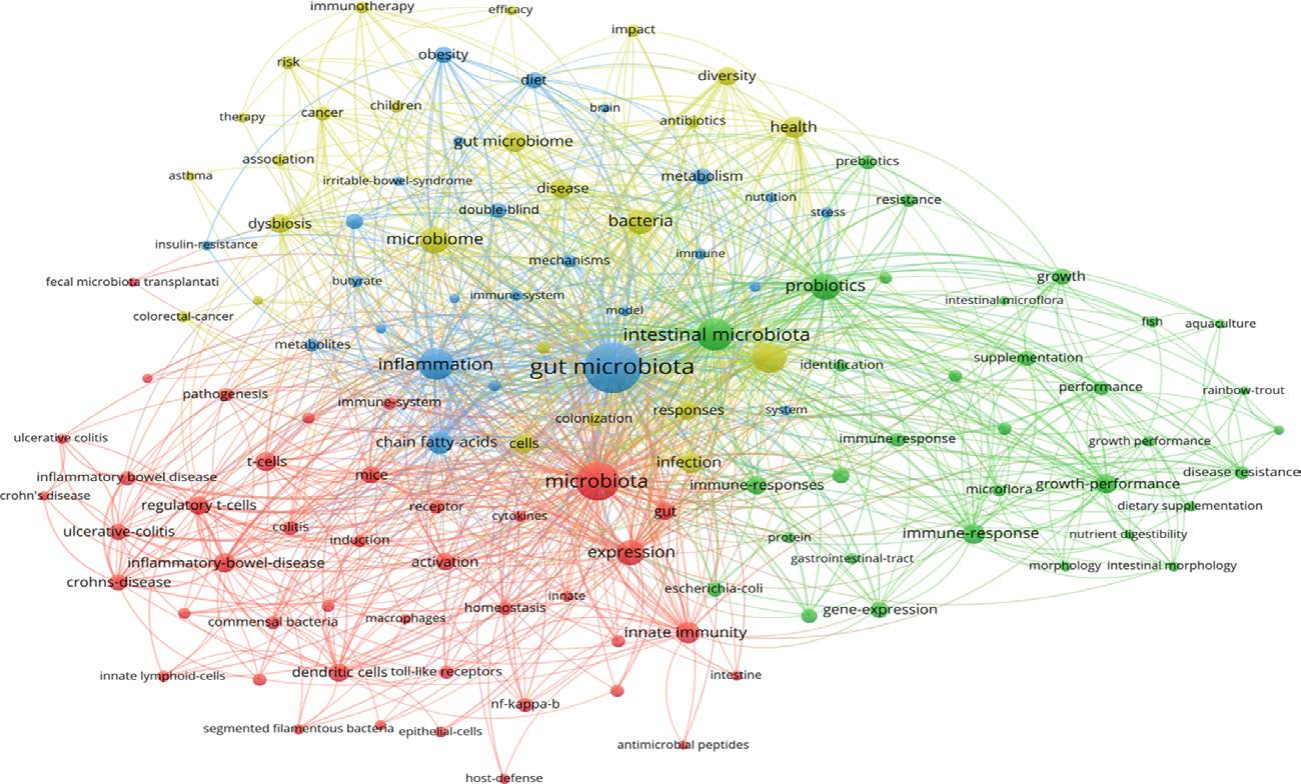
Visualization of keyword co-occurrence analysis. The size of nodes indicates the frequency of occurrences of the keywords. The lines between the nodes represents their co-occurrence in the same publication. The shorter the distance between 2 nodes, the larger the number of co-occurrence of the 2 keywords.

The network visualization diagram of keywords is shown in Figure 7, with 4 clusters in red, green, yellow, and blue indicating 4 research directions. The green cluster includes keywords such as gut microbiota, probiotics, immune response, gene expression, and gastrointestinal tract. The blue cluster includes keywords such as gut microbiota microorganisms, inflammation, obesity, diet, metabolism, and immune system. The red cluster includes keywords such as microbiota, expression, innate immunity, activation, inflammatory bowel disease, T cells, regulation of T cells, etc. The yellow cluster includes keywords such as microbiome, gut microbiome, bacteria, health, diversity, response, infection, cells, ecological imbalance, immunotherapy, etc.

## 4. Discussion

### 4.1. General information

This article presents a bibliometric analysis of the field of gut microbiota and immunity. In our study, a total of 16,611 publications were included, consisting of 10,865 articles and 5746 reviews. Since 2013, the number of publications in this field has increased rapidly. A total of 62,872 scholars have contributed to this field. The top 10 scholars with the highest publication output are all from China. All publications came from 2144 journals and were published by approximately 9965 organizations in 145 countries/regions. The leading organizations are UNIVERSITY OF CALIFORNIA SYSTEM, UDICE FRENCH RESEARCH UNIVERSITIES and HARVARD UNIVERSITY. The top 3 countries with the highest publication output are the United States, the People Republic of China, and Italy. The top 3 journals with the highest publication output are FRONTIERS IN IMMUNOLOGY, FRONTIERS IN MICROBIOLOGY, and PLOS ONE. The first highly cited paper was published in Science and has been cited 3006 times by other studies, with authors including Patrick M. Smith, Michael R. Howitt, and Nicolai Paniko. Four indicative research directions were identified: green clustering keywords include gut microbiota, probiotics, immune response, gene expression, and gastrointestinal tract; blue clustering keywords include gut microbiota microorganisms, inflammation, obesity, diet, metabolism, and immune system; red clustering keywords include microbiota, expression, innate immunity, activation, inflammatory bowel disease, T cells, and regulation of T cells; yellow clustering keywords include microbiome, gut microbiome, bacteria, health, diversity, response, infection, cells, ecological imbalance, and immunotherapy.

In this bibliometric analysis, the majority of articles came from the United States (29%; 4900/16611), the People Republic of China (27%; 4547/16611), and Italy (6.4%; 1063/16611). Among the top 10 institutions with the highest publication output, 4 are from France, 3 are from the United States, 2 are from China, and one is from the United Kingdom. The top 10 scholars with the highest publication output are all from China. In the field of gut microbiota and immunity, the most prominent contributions come from France, the United States, and China. This indicates that research efforts in other countries need to be strengthened.

### 4.2. Hotspots and frontiers

Based on the analysis of keyword co-occurrence clustering and highly cited literature, the research hotspots and frontiers are as follows.

#### 4.2.1. Gut microbiota and inflammation.

Among the top 10 most frequently cited articles, 5 articles discussed the relationship between gut microbiota and inflammation, with the keywords “gut microbiota” and “inflammation” located in the blue cluster. Regulatory T cells expressing transcription factor Foxp3 are crucial for gut inflammation, and Smith et al have demonstrated that they are modulated by specific bacterial strains in mouse models. Numerous research hotspots focus on how gut microbiota regulate the balance of Treg cells.^[35–38]^

Patrick M. Smith1, Michael R. Howitt1, Nicolai Paniko et al^[31]^discovered that bacterial fermentation products derived from gut microbiota – short-chain fatty acids, can regulate the size and function of the colonic Treg pool through G protein-coupled receptor 43(GPCR43) mediation expressed in the colon, and protect mice from colitis in a Free fatty acid receptor 2 (Ffar2)-dependent manner. Their study indicates that a class of abundant microbial metabolites is the basis for the co-adaptation of the adaptive immune microbiota and promotes colonic homeostasis and health. Arpaia, N et al^[33]^ also demonstrated through mouse experiments that bacterial metabolites mediate communication between the symbiotic microbiota and the immune system, affecting the balance between pro-inflammatory and anti-inflammatory mechanisms. Additionally, other researchers^[32]^ found that the gut symbiont Clostridium, can shape the mucosal immune system by regulating the differentiation and expansion of several T cells, thereby intervening in inflammation and allergy reactions.

In conclusion, manipulating gut microbiota holds promise for the treatment of inflammatory and allergic diseases. Many probiotic microorganisms have been identified, but there is still an urgent need to discover specific strains that can elicit stronger therapeutic responses, be compatible with the host, and influence the host immune system in a well-controlled physiological manner.

#### 4.2.2. Gut microbiota and immune response.

Among the top 10 highly cited articles, 9 of them investigate the relationship between gut microbiota and immune response, with the keywords related to gut microbiota and immune response belonging to the green cluster. The microbiota plays a critical role in inducing, training, and shaping the host immune system. In return, the immune system has evolved as a means to maintain a symbiotic relationship with these highly diverse and continuously evolving microorganisms. When functioning optimally, this immune-microbiota alliance can induce protective responses against pathogens and maintain regulatory pathways involved in maintaining tolerance to harmless antigens. However, in some high-income countries, the excessive use of antibiotics, changes in dietary patterns, and elimination of compositional partners (such as helminths) may select for a dysbiotic microbiota lacking the resilience and diversity required to establish balanced immune responses, thereby impacting the normal function of the immune system.

Overall, the research on the interaction between gut microbiota and immune response indicates the crucial role of microbiota in immune regulation and highlights the potential consequences of dysbiosis on immune system functioning. Understanding and manipulating the gut microbiota to promote a balanced and beneficial immune response hold significant implications for maintaining overall health and preventing immune-related disorders.

#### 4.2.3. Microbiota and inflammatory bowel disease.

Among the top 10 highly cited articles, 2 of them investigate the relationship between microbiota and IBD, with the keywords related to microbiota and IBD belonging to the red cluster. IBD refers to a group of nonspecific chronic gastrointestinal inflammatory diseases, including ulcerative colitis (UC), Crohn disease (CD), and indeterminate colitis (IC), with unknown etiology and pathogenesis. The current understanding suggests that the pathogenesis of IBD involves dysregulated intestinal mucosal immune response triggered by an imbalanced gut microbiota in genetically susceptible individuals, leading to intestinal mucosal damage. Genetic pathways associated with the host imply the potential role of abnormal immune responses to gut microbiota. Dirk Gevers et al^[30]^ conducted a comprehensive analysis of the microbiota from multiple gastrointestinal sites collected before treatment in newly diagnosed pediatric CD patients. They found that the axis defined by increased abundance of bacterial families including Enterobacteriaceae, Pasteurellaceae, Veillonellaceae, and Clostridiaceae, and decreased abundance in the orders Enterobacteriales, Burkholderiales, and Clostridiales, was closely associated with the disease state. A comparison of microbiota between CD patients with and without antibiotic exposure indicated that antibiotic use exacerbated the microbial dysbiosis associated with CD. Comparisons of microbial features among samples from the ileum, rectum, and feces suggested that assessing rectal mucosa-associated microbiota holds unique potential for convenient and early diagnosis of CD in the early stages of the disease.

The research on the relationship between microbiota and inflammatory bowel disease provides valuable insights into the pathogenesis of IBD and highlights the importance of maintaining a balanced gut microbiota for intestinal health. Understanding the mechanisms underlying microbiota dysbiosis and its impact on immune responses in IBD can contribute to the development of novel therapeutic strategies and interventions for the management of IBD.

#### 4.2.4. Microbiota and cancer treatment.

Among the top 10 most frequently cited articles, 2 studies have investigated the relationship between microbiota and tumors, with the keywords pertaining to microbiota and tumors falling within the yellow cluster. The impact of gut microbiota on the release of immune-mediated anti-tumor T cell responses has heralded a new era in cancer therapy. While these treatments significantly contribute to tumor regression in certain patients, many others derive no benefit. Some scholars posit^[39]^ that gut probiotics aid in combating cancer, as the presence of resident gut bacteria influences patients’ response to cancer immunotherapy. Routy et al^[25]^ have shown a correlation between antibiotic usage and adverse reactions to immune therapy involving PD-1 inhibitors. By analyzing samples from patients with lung and kidney cancer, they discovered lower levels of Akkermansia muciniphila bacteria in non-responsive individuals. Restoring the response to immune therapy was achieved by orally supplementing mice treated with antibiotics with additional bacteria. In their research on melanoma patients receiving PD-1 inhibitors, Matson, Gopalakrishnan et al^[26]^ found that responding patients harbored a greater abundance of “beneficial” bacteria in their intestines. Non-responders exhibited an imbalanced composition of gut microbiota, which correlated with impaired immune cell activity. Scholars have identified specific members of the gut microbiota that impact the efficacy of such immune therapies in mouse animal experiments.^[27]^ They discovered that optimal response to anti-cytotoxic T lymphocyte antigen blockade necessitated the presence of specific Bacteroides species. Similarly, Sivan et al^[27]^ identified the genus Bifidobacterium, which enhanced the efficacy of anti-programmed cell death ligand 1 therapy. The gut microbiota modulates the effectiveness of cancer immunotherapy in mice. T cell infiltration into solid tumors correlates with favorable patient prognosis, yet the mechanisms underlying variable immune responses among individuals remain unclear. One possible modulator could be the gut microbiota. Experiments comparing melanoma growth in mice with different cohabitating microbiota revealed differences in spontaneous anti-tumor immunity, which were eliminated through co-housing or fecal transfer. 16S ribosomal RNA sequencing identified Bifidobacterium as being associated with anti-tumor effects. Administration of Bifidobacterium alone orally improved tumor control to the same degree as treatment with PD-L1-specific antibody therapy (checkpoint blockade), while combination therapy nearly eradicated tumor growth. Enhanced dendritic cell function led to heightened activation of CD8 + T cells and their accumulation within the tumor microenvironment, mediating this effect. Relevant data suggests that manipulation of the microbiota may regulate cancer immunotherapy. Maintaining a healthy gut microbiota can aid patients in combating cancer.

### 4.3. Strengths and limitations

This study represents the first bibliometric analysis of the gut microbiota and immunity. In our research, we have presented the current state of the field, created visual maps, and explored trends and hot topics, providing a reference for future investigations. However, our study has several limitations. Firstly, all data were extracted solely from WoSCC, without incorporating other databases like Medline and Embase. Secondly, only studies published in English were included. Lastly, despite employing standardized procedures, biases may still exist. Further research will offer new insights into this field.

## 5. Conclusion

The main research hotspots in the field of gut microbiota and immunity are gut microbiota and inflammation, gut microbiota and immune response, microbiota and inflammatory bowel disease, and microbiota and tumors. The stability of gut microbiota and its microorganisms plays an important role in regulating the immune response, inflammation, and even tumors and inflammatory bowel diseases. Previous studies have generated impressive data showing the impact of gut microbiota and its microorganisms on maintaining human health, immune, inflammatory, and oncogenic responses. Autoimmune and inflammatory diseases are associated with immune dysfunction, which has been rapidly increasing in the past few decades. Immuno-oncology has become a hot field with the development of immune checkpoint inhibitors. In recent years, immunology research has shifted from a lymphoid-centric view of the immune system to an understanding of the tissue microenvironment as the fundamental determinant of immune responses. This area of research has led to the integration of the microbiota as an endogenous regulator of all immune responses. Currently, with increasing knowledge of the role of microbiota composition, key bacterial species, and their metabolites or derivatives, especially the connection between some of these components and human disease states, there has been a wealth of discoveries. This provides scientists and clinicians with a unique opportunity to explore human health from multiple perspectives including ecology, nutrition, genetics, microbiology, biochemistry, and immunology. In this multidisciplinary field of research, the key lies in manipulating or restoring the immunological-microbial dialogue to promote or restore the health of the human meta organism.

## Author contributions

**Conceptualization:** Luming Qi.

**Data curation:** Kaidi Nie, Nannan LIU.

**Formal analysis:** Kaidi Nie, Jie Wang, Zhixuan Chen.

**Investigation:** Tingting Deng, Nannan LIU, Zhixuan Chen.

**Methodology:** Lina Xia, Jie Wang, Nannan LIU.

**Supervision:** Kaidi Nie, Lina Xia.

**Software:** Luming Qi.

**Validation:** Tingting Deng.

**Visualization:** Jie Wang, Luming Qi, Zhixuan Chen.

**Writing – original draft:** Kaidi Nie.

**Writing – review & editing:** Kaidi Nie, Lina Xia, Jie Wang.
